# Design and Implementation of Miniaturized Low-Frequency Flexibility-Enhanced Rotating Cantilever Beam Piezoelectric MEMS Microphone

**DOI:** 10.3390/mi17040488

**Published:** 2026-04-17

**Authors:** Bingchen Wu, Gong Chen, Changzhi Zhong, Tao Wang

**Affiliations:** 1 College of Communication Engineering, Chengdu University of Information Technology, Chengdu 610225, China; wbc_cuit@foxmail.com (B.W.);; 2 Chimesen Technology Co., Ltd., Chengdu 610095, China

**Keywords:** Piezoelectric MEMS Microphone (PMM), triangular cantilever beam, flexibility-enhanced rotating cantilever beam, scandium-doped aluminum nitride (AlScN), signal-to-noise ratio (SNR)

## Abstract

In response to the pressing need for miniaturized MEMS microphones in wearable technology and mobile devices, and to surmount the technical limitations inherent in conventional piezoelectric microphones, which typically depend on enlarging chip dimensions or decreasing stiffness to attain low resonance frequencies, this study introduces a novel piezoelectric MEMS microphone (PMM) design predicated on a flexibility-enhanced rotating structure. The proposed design utilizes an aluminum scandium nitride (Al_0.8_Sc_0.2_N) piezoelectric thin film with 20% scandium doping and incorporates four equivalent sensing units formed by four curved cutting lines centrally located on the chip. This configuration employs a nested arrangement of four cantilever beams to substantially increase vibration compliance, thereby effectively lowering the natural frequency without altering the chip’s external size. Three-dimensional finite element simulations reveal that, relative to traditional triangular cantilever beam architectures, the flexibility-enhanced rotating structure reduces the natural frequency from 15.6 kHz to 13.49 kHz while enhancing sensitivity from −44.6 dB to −40 dB. The device was fabricated via a comprehensive microfabrication process and subsequently characterized within a standardized acoustic testing environment. Experimental results indicate that the microphone attains a sensitivity of −43.84 dB at 1 kHz and exhibits a first resonance frequency of 13.5 kHz, closely aligning with simulation predictions. Furthermore, the signal-to-noise ratio (SNR) reaches 58.3 dB across the full range of human-audible frequencies. By leveraging the flexibility-enhanced rotating structure, this work achieves an optimal compromise between elevated sensitivity and reduced resonance frequency within a compact form factor, thereby offering a viable technical solution for the advancement of high-performance miniature acoustic sensors.

## 1. Introduction

AlN has emerged as the preferred material for Piezoelectric MEMS Microphones (PMMs) owing to its full compatibility with CMOS/MEMS processes, low dielectric constant, minimal energy loss, and high mechanical quality factor. Empirical studies have demonstrated AlN’s exceptional performance, characterized by wide bandwidth, low power consumption, and high SNR [1,2,3]. Building upon this foundation, researchers have enhanced the piezoelectric properties by alloying AlN with scandium (Sc), forming AlScN compounds. When the scandium concentration reaches approximately 40–45%, the piezoelectric coefficient d_33_ increases by a factor of about five relative to pure AlN. Although this enhancement is accompanied by a reduction in the quality factor (Q), the resultant improvement in sensitivity is notably significant [2,4]. Concurrently, the implementation of double-layer or multilayer AlN cantilever beam structures has been shown to effectively reduce the noise floor and augment output performance [3,5,6,7,8]. Despite these advances, current PMM designs predominantly achieve low resonance frequencies by either enlarging the chip size or decreasing the stiffness of the vibrating elements [6,9,10]. Such approaches result in increased device volume, thereby impeding the miniaturization imperative for wearable technologies and mobile devices. To address limitation, the present study introduces an innovative diaphragm architecture based on Al_0.8_Sc_0.2_N piezoelectric thin films. This design incorporates flexibility-enhanced rotating cantilever beams and optimizes geometric configurations while maintaining constant overall chip dimensions. By substantially increasing vibration compliance, the proposed structure attains lower resonance frequencies, thereby enhancing sensitivity under a standardized sound pressure of 1 Pa. This approach effectively balances compact device size with low-frequency responsiveness, offering a viable technical solution for the practical deployment of PMMs in applications such as smart wearables, mobile devices, and miniature acoustic sensors.

## 2. Simulation Design of Flexibility-Enhanced Rotating Cantilever Beam PMM

**Figure 1:**
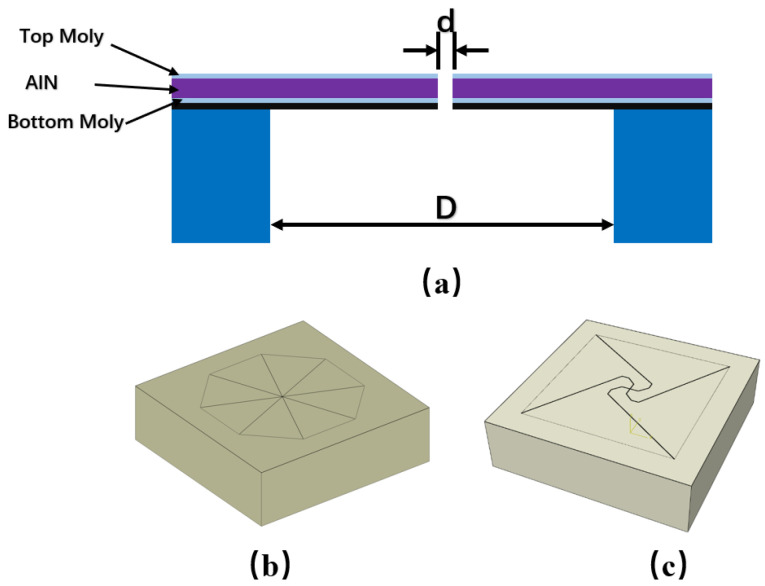
(**a**) Sectional diagram of the cantilever beam PMM; (**b**) Typical triangular cantilever beam PMM; (**c**) Flexibility-enhanced rotating cantilever beam PMM.

At present, the prevailing cantilever configuration for PMMs primarily utilizes triangular cantilever beams [7,8,11], which have become the conventional design within this domain owing to their advantageous mechanical characteristics and straightforward fabrication process, as illustrated in Figure 1b. In order to further decrease the natural frequency while preserving the external dimensions of the chip, this study introduces an innovative flexibility-enhanced rotating PMM, depicted in Figure 1c. The fundamental concept of this design involves the creation of four equivalent sensing units through the implementation of four curved cutting lines at the center of the chip, with the apexes of the four cantilever beams interlocked.

The fundamental mechanism focuses on enhancing structural compliance through the synergistic deformation of multiple units to achieve a reduction in natural frequency without increasing the chip footprint. Unlike conventional triangular cantilevers, which are constrained to a single bending mode, the proposed design incorporates four nested equivalent sensing units created by curved cutting lines at the chip’s center. This configuration allows for independent bending and the superposition of deformations at the nested nodes, thereby substantially increasing overall compliance without modifying the chip’s dimensions. The implementation of curved cutting lines serves to optimize the distribution of moment arms, reduce stress concentrations, and enlarge the effective vibration area. Additionally, a gradient stiffness distribution—characterized by low stiffness in the central vibration region and high stiffness in the peripheral support region—ensures mechanical stability while enhancing compliance, thereby preventing structural failure commonly associated with the overall stiffness reduction observed in traditional designs.

**Figure 2:**
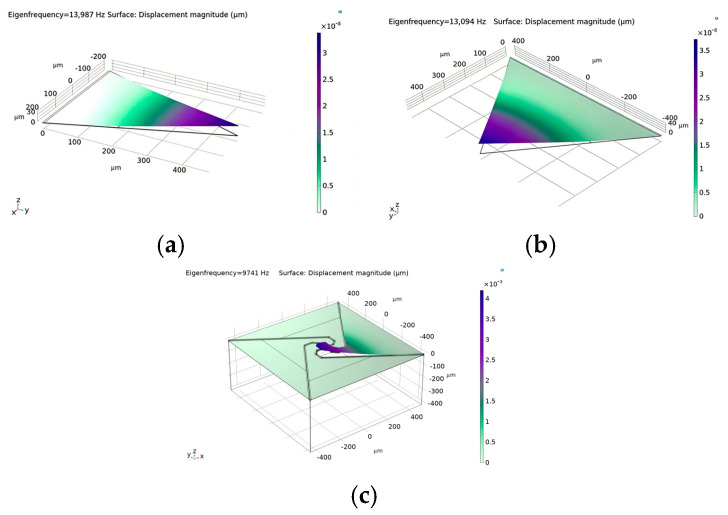
(**a**) Natural frequency FEM results of triangular cantilever beam; (**b**) Natural frequency FEM results of increased-area triangular cantilever beam; (**c**) Natural frequency FEM results of flexibility-enhanced rotating cantilever beam.

Utilizing three-dimensional finite element analysis with identical cavity dimensions, this study compares the natural frequencies of conventional triangular cantilever beams to those of a novel structural design. The findings substantiate the enhanced frequency modulation capability of the flexibility-augmented rotating structure. As illustrated in Figure 2, merely enlarging the effective film area of the traditional triangular cantilever beam results in a modest frequency reduction from 13.99 kHz to 13.09 kHz. In contrast, implementing the proposed four-beam nested compliance configuration leads to a pronounced frequency decrease to 0.97 kHz. This substantial reduction is attributed to the marked enhancement in overall structural compliance afforded by the four equivalent sensing unit design, which enables directional stiffness regulation without increasing the chip footprint. These results suggest that, within constrained chip dimensions, the flexibility-enhanced rotating structure affords greater design flexibility for the film, permitting precise control of the natural frequency through adjustments in the supporting beam geometry [8,12,13]. The lowered natural frequency improves the device’s compatibility with low-frequency acoustic signals, facilitates more uniform and comprehensive participation of the piezoelectric film in energy conversion, and consequently enhances sensitivity within the human-audible frequency range. This demonstrates superior acoustic performance, particularly in applications demanding elevated signal-to-noise ratios, such as smart wearable devices and mobile terminals.

**Figure 3:**
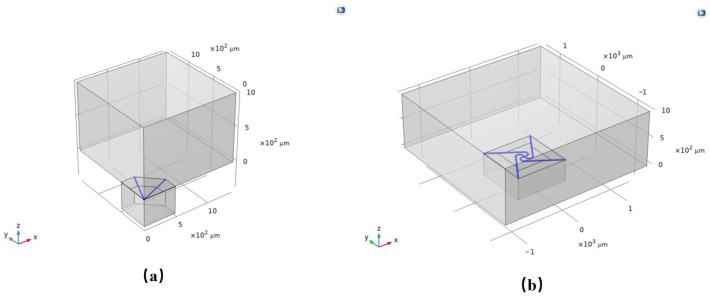
(**a**) Simulation model of a triangular cantilever beam PMM with an acoustic channel; (**b**) Simulation model of a flexibility-enhanced rotating cantilever beam PMM with an acoustic channel.

To systematically assess the benefits of the proposed flexibility-enhanced rotating cantilever beams relative to conventional triangular cantilever beams, this study models and analyzes the two chip configurations depicted in Figure 1b,c under identical simulation conditions. Owing to its full planar symmetry, the conventional triangular cantilever beam is represented using a one-quarter symmetric model to improve computational efficiency. In contrast, the centrally symmetric flexibility-enhanced rotating structure necessitates the use of a full model for analysis, as illustrated in the schematic presented in Figure 3.

**Figure 4:**
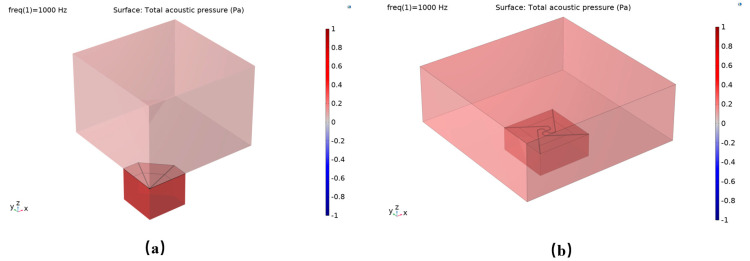
(**a**) Simulated sound pressure distribution of a triangular cantilever beam PMM with an acoustic channel; (**b**) Simulated sound pressure distribution of a flexibility-enhanced rotating cantilever beam PMM with an acoustic channel.

**Figure 5:**
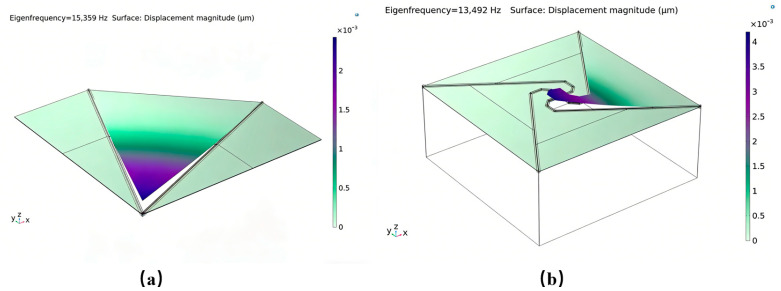
(**a**) Natural frequency FEM results of a triangular cantilever beam PMM with an acoustic channel; (**b**) Natural frequency FEM results of a flexibility-enhanced rotating cantilever beam PMM with an acoustic channel.

**Figure 6:**
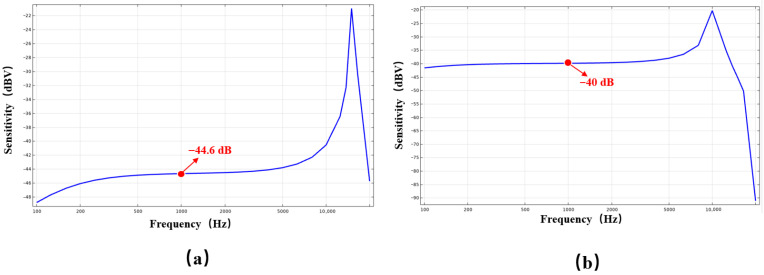
(**a**) Simulated frequency response of a triangular cantilever beam PMM with an acoustic channel; (**b**) Simulated frequency response of a flexibility-enhanced rotating cantilever beam PMM with an acoustic channel.

To examine the impact of acoustic channels on overall device performance, simulations were conducted incorporating identical acoustic channels on both chip designs, with comprehensive model parameters detailed in Table 1. The simulation outcomes indicate the following: (1) The sound pressure distribution (Figure 4) remains largely unchanged for both chips upon the addition of acoustic channels, suggesting that the channels exert a consistent effect on the sound field distribution across both configurations; (2) Analysis of the natural frequency under uniform boundary conditions (Figure 5) reveals that the reference structure exhibits a resonance frequency of 15.6 kHz, whereas the compliance structure demonstrates a reduced resonance frequency of 13.49 kHz, thereby confirming that the centrally symmetric four-beam nested design effectively lowers the resonance frequency; (3) The sensitivity–frequency response (Figure 6) further illustrates performance improvements, with the reference structure showing a sensitivity of −44.6 dB and the compliance structure achieving an enhanced sensitivity of −40 dB, representing an increase of approximately 5.4 dB, which corresponds to nearly double the original sensitivity.

In summary, by preserving the chip’s external dimensions, the proposed flexibility-enhanced rotating PMM attains a reduced natural frequency and increased sensitivity through its centrally symmetric four-beam nested configuration. This design exhibits notable improvements in overall acoustic performance relative to conventional triangular cantilever beam structures, thereby offering a viable technical approach for the miniaturization and low-noise optimization of PMMs.

## 3. Manufacturing of Flexibility-Enhanced Rotating Cantilever Beam PMM

**Figure 7:**
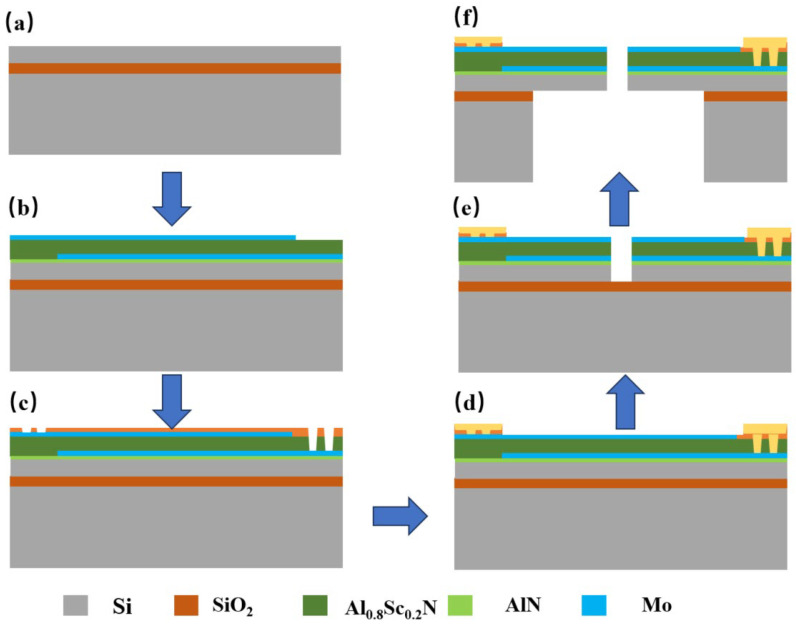
Fabrication process flow of the PMM. (**a**) SOI substrate preparation; (**b**) Mo/Al_0.8_Sc_0.2_N stack deposition and patterning; (**c**) Via holes etching for electrode interconnection; (**d**) Signal metal electrode definition; (**e**) Cantilever gap structure etching; (**f**) Beam release by backside DRIE.

The fabrication procedure for the proposed flexibility-enhanced rotating cantilever beam PMM is depicted in Figure 7. Initially, a customized silicon-on-insulator (SOI) wafer is selected as the substrate to enable precise regulation of the piezoelectric film thickness (Figure 7a). Subsequently, physical vapor deposition (PVD) is employed to sequentially deposit a molybdenum (Mo) electrode layer followed by an Al_0.8_Sc_0.2_N piezoelectric thin film. The Mo electrode serves to provide lattice matching compatible with Al_0.8_Sc_0.2_N, thereby ensuring the crystalline integrity of the piezoelectric layer [14]. Following the deposition of the piezoelectric film, reactive ion etching (RIE) is utilized to pattern the Mo electrode, forming the electrode–piezoelectric stack structure as illustrated in Figure 7b [15]. Thereafter, Al_0.8_Sc_0.2_N etching (RIE) is performed to create via holes within this stack, facilitating electrical interconnection between the upper and lower electrodes (Figure 7c) [16]. The aperture dimensions are meticulously controlled to prevent electrical signal instability resulting from Mo over-etching. Next, photolithography and etching processes are applied to the aluminum/copper metal layer to define metal electrodes for signal extraction (Figure 7d) [17]. Subsequently, the gap structure of the cantilever beams is etched into the silicon layer, with precise adjustment of the gap width effectively mitigating the formation of microphone leakage channels (Figure 7e) [12,18]. Finally, deep reactive ion etching (DRIE) is employed to release the cantilever beams by removing the silicon bulk and thermal oxide layer from the wafer’s backside, culminating in a fully released cantilever structure capable of free vibration under acoustic pressure excitation (Figure 7f) [19,20]. This fabrication approach yields a high-compliance, low-resonance-frequency PMM structure while preserving the overall chip dimensions, thereby providing a robust manufacturing strategy for miniaturized acoustic sensing devices.

**Figure 8:**
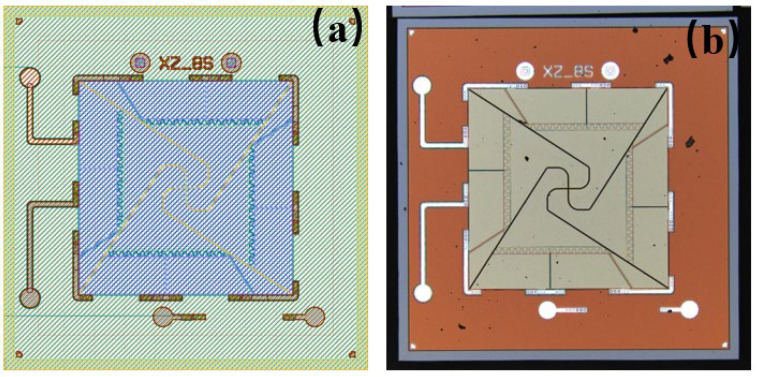
(**a**) Layout of the fabricated chip; (**b**) Microscope image of the fabricated chip.

Figure 8 illustrates the device layout of the flexibility-enhanced rotating cantilever beam PMM as well as a microscope image of the PMM device fabricated using the previously described process.

## 4. Performance Evaluation of the Flexibility-Enhanced Rotating Cantilever Beam PMM

**Figure 9:**
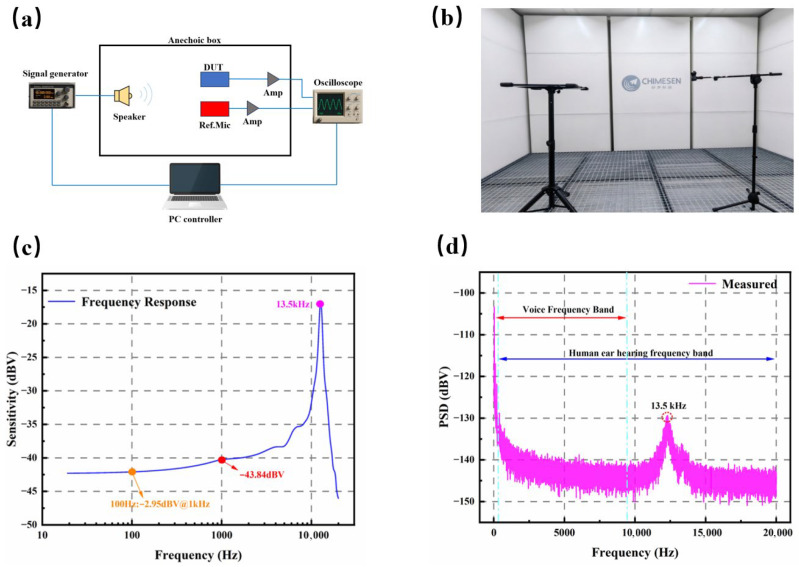
(**a**) Simplified schematic of electroacoustic measurement setup; (**b**) The frequency response acoustic experimental environment; (**c**) The frequency response diagram of PMM; (**d**) The measured noise diagram of PMM.

To evaluate the acoustic performance of the fabricated microphone, a comprehensive experimental setup was developed and thoroughly characterized [21,22]. All measurements were performed within a specially designed small anechoic chamber, the internal configuration of which is illustrated in Figure 9a.

The experimental arrangement employed a symmetrical configuration wherein the Device Under Test (DUT) and a reference microphone (GRAS 46AE, The GRAS 46AE microphone was supplied by GRAS Sound & Vibration A/S, Holte, Denmark) were placed equidistantly at 30 cm from the reference speaker (KEF Q100, The KEF Q100 loudspeaker was acquired from KEF Audio, Maidstone, Kent, UK) to ensure consistent acoustic excitation. Rigorous environmental controls were enforced to reduce measurement artifacts. The chamber walls were entirely covered with sound-absorbing foam to suppress reflections, while ambient temperature and relative humidity were maintained at 25 ± 1 °C and 50 ± 5%, respectively, to ensure measurement stability and accuracy.

The calibration procedures were conducted in strict accordance with international acoustic measurement standards. Initially, the output sound pressure level (SPL) and frequency response of the source loudspeaker (KEF Q100) were meticulously calibrated utilizing a sound calibrator (BRUEL & KJER 4231, 94 dB at 1 kHz, The BRUEL& KJER 4231 calibrator was obtained from Bruel & Kjer Sound & Vibration Measurement A/S, Nærum, Denmark). This calibration was performed in triplicate to minimize random errors, with the mean value subsequently employed for further analyses. Following the speaker calibration, the established sound field and a reference microphone (GRAS 46AE) were employed to verify the integrity of the entire measurement system. The system was considered acceptable only if the discrepancy between the measured and reference SPL values was less than 0.5 dB.

In the signal acquisition system, the raw signals obtained from both the device under test (DUT) and the reference microphone (GRAS 46AE) were initially amplified using low-noise preamplifiers characterized by a gain of 20 dB and a bandwidth ranging from 20 Hz to 20 kHz. This amplification served to enhance weak signals while minimizing noise interference. Subsequently, the amplified signals were transmitted to a data analyzer (Keysight N9020B) for real-time acquisition and analysis. The data acquisition was conducted at a sampling rate of 100 kHz with a duration of 10 s per frequency point, thereby ensuring an adequate dataset for the accurate computation of sensitivity, noise spectral density, and total harmonic distortion (THD).

The experimental results are presented in Figure 9c. At a frequency of 1 kHz, the measured microphone sensitivity was recorded at −43.84 dBV, which closely aligns with the reference target of −40 dBV established based on prior simulation design. Taking into account manufacturing process variations, this outcome substantiates the efficacy of the design and demonstrates the achievement of high sensitivity within a miniaturized form factor. The device’s fundamental resonance frequency was measured at 13.5 kHz, exhibiting strong agreement with the simulated value of 13.49 kHz. This concordance not only validates the feasibility of the proposed mechanical structure in attaining a reduced resonance frequency within constrained dimensions but also ensures that, since this resonance frequency significantly exceeds the voice frequency range (20 Hz to 8 kHz) [11], the device maintains a smooth frequency response throughout the operational bandwidth. Furthermore, under a 1 kHz excitation frequency and a sound pressure level of 94 dB SPL (1 Pa), the total harmonic distortion (THD) was measured to be below 0.1%. The frequency response curve indicates that the roll-off frequency, defined as the point where sensitivity decreases by 3 dB relative to the 1 kHz reference, occurs near 100 Hz [23]. This performance is attributed to the effective regulation of diaphragm deformation afforded by the structural design.

The noise performance of the device was evaluated within an anechoic chamber environment to effectively eliminate external environmental interference [24,25,26]. The noise floor was directly measured using data acquisition software, with the results presented in Figure 9d. The spectral analysis reveals a predominance of low-frequency noise, primarily attributed to the challenges in fully shielding against environmental disturbances. A local peak in the noise spectral density is observed near the first resonance frequency at 13.5 kHz, which aligns with the expected increase in thermo-mechanical noise due to mechanical resonance. Across the entire human-audible frequency range (20 Hz to 20 kHz), the calculated A-weighted noise level is −102 dBA, corresponding to a signal-to-noise ratio (SNR) of 58.3 dB. Within the voice frequency band (20 Hz to 8 kHz), the A-weighted noise level slightly rises to −100 dBA, with an improved SNR of 60.5 dB. The analysis indicates that low-frequency noise constitutes the primary component of the noise floor, whereas noise arising from component resonance has a comparatively minor impact on the overall noise level [27,28,29]. Ensuring an adequate SNR is essential for maintaining data accuracy, particularly in measurements involving low signal levels [11].

Table 2 presents a comparative analysis of the key performance parameters of the PMM are based on the flexibility-enhanced rotating structure proposed in this study are against those reported in similar research. The results indicate that the microphone developed herein exhibits notable advantages in overall performance. Despite having a diaphragm size (0.91 mm^2^) comparable to or slightly exceeding that of related works, the present design attains an unamplified sensitivity of −43.8 dB, which is substantially higher than most of the referenced devices. Additionally, its SNR of 58.3 dB surpasses the majority of studies employing similar Sc-doped AlN materials and aligns with the performance level of recent high-performance MEMS microphones. Although the study by A. Rahaman et al. reported a superior SNR of 67 dB, their diaphragm area (4.9 mm^2^) is considerably larger than that of the current design [12]. In summary, this work successfully achieves an optimal balance between elevated sensitivity and favorable SNR within a compact form factor, thereby demonstrating the efficacy of the proposed flexibility-enhanced rotating structure.

## 5. Conclusions

This study addresses the challenges of miniaturization and low-noise requirements of PMMs by introducing and fabricating an innovative diaphragm design based on flexibility-enhanced rotating cantilever beams. The design incorporates four curved cutting lines centrally arranged on the chip, forming four equivalent sensing units that collectively create a nested four-beam structure with enhanced flexibility. This configuration reduces the natural frequency from 13.99 kHz, typical of conventional triangular cantilevers, to 0.97 kHz without altering the chip dimensions, thereby significantly extending the device’s low-frequency response range. Microfabrication processes based on SOI wafers, including PVD, RIE, and DRIE, were employed to achieve the deposition and patterning of Mo/Al_0.8_Sc_0.2_N multilayer films and to release the cantilever beams, ensuring both high flexibility and structural reliability. Experimental characterization revealed a sensitivity of −43.8 dBV at 1 kHz and a first resonance frequency of 13.5 kHz, closely matching simulation results. The device exhibited a total harmonic distortion (THD) below 0.1%, an A-weighted noise level of −102 dBA across the full frequency spectrum, and −100 dBA within the voice frequency band, corresponding to SNRs of 58.3 dB and 60.5 dB, respectively, indicating excellent noise suppression capabilities. Compared to existing MEMS microphones employing similar materials or larger diaphragm areas, this design achieves superior sensitivity and SNR within a compact footprint of only 0.91 mm^2^, demonstrating the significant advantages of the flexibility-enhanced rotating cantilever structure in optimizing the trade-off between device size and performance.

Notwithstanding these findings, the present study exhibits certain limitations. The experimental frequency spectra reveal a notable presence of low-frequency noise, which predominantly arises from challenges associated with fully mitigating environmental disturbances. Furthermore, while scandium (Sc) doping significantly improves the piezoelectric coefficient, it concurrently results in a reduction in the mechanical quality factor (Q). This trade-off may restrict the applicability of the material in specific contexts that demand devices with high-frequency performance or elevated Q values.

Future research will concentrate on optimizing the packaging architecture to reduce the coupling effects of low-frequency environmental noise. Additionally, investigations will be conducted into innovative material systems and composite structural designs. The objective is to restore or improve the quality factor without compromising sensitivity, thereby promoting the advancement of high-performance miniature acoustic sensors for practical applications.

## Figures and Tables

**Table 1:** Structural Dimensions for FEM Simulation.

Parameter	Parameter Symbol	Value
Chip Al_0.8_Sc_0.2_N Thickness	T1	500 μm
Chip Top Electrode Thickness	T2	60 μm
Chip Bottom Electrode Thickness	T3	60 μm
Chip Back Cavity	D	750 μm
Chip Slit Width	d	2 μm
Package Dimensions	L × W × H	3.76 mm × 2.95 mm × 1.2 mm

**Table 2:** Summary of the Main Parameters in the PMMS.

Author	Piezoelectric Material	Diaphragm Size (mm^2^)	Unamplified Sensitivity (dB)	SNR at 1 kHz (dB)
Rahaman, A. et al. [12]	AlN	4.9	− 46.9	67
Williams, M.D. et al. [13]	AlN	0.54	− 88.2	54
Hu, B. et al. [7]	Al_0.905_Sc_0.095_N	0.5	− 73.7	54.2
Yan, R. et al. [11]	Bimorph Al_0.8_Sc_0.2_N	0.81	− 55.5	54.5
This work	Unimorph Al_0.8_Sc_0.2_N	0.91	−43.8	58.3

## Data Availability

The original contributions presented in the study are incorporated within the article. For further inquiries, please direct them to the corresponding author.
